# In Search of a New Paradigm for Functional Magnetic Resonance Experimentation With Language

**DOI:** 10.3389/fneur.2020.00588

**Published:** 2020-06-24

**Authors:** Maria Eugênia Arantes, Fernando Cendes

**Affiliations:** ^1^Faculty of Medical Sciences, University of Campinas—UNICAMP, Campinas, Brazil; ^2^Laboratory of Neuroimaging, Department of Neurology, University of Campinas—UNICAMP, Campinas, Brazil

**Keywords:** language, fMRI, neuroimaging, neurolinguistics, experimental linguistics, methodology

## Abstract

Human language can convey a broad range of entities and relationships through processes that are highly complex and structured. All of these processes are happening somewhere inside our brains, and one way of precising these locations is through the usage of the functional magnetic resonance imaging. The great obstacle when experimenting with complex processes, however, is the need to control them while still having data that are representative of reality. When it comes to language, an interactional phenomenon in its nature, and that integrates a wide range of processes, a question emerges concerning how compatible it is with the current experimental methodology, and how much of it is lost in order to fit the controlled experimental environment. Because of its particularities, the fMRI technique imposes several limitations to the expression of language during experimentation. This paper discusses the different conceptions of language as a research object, the hardships of combining this object with the requirements of fMRI, and what are the current perspectives for this field of research.

## Introduction

Attempts to discover the dwelling of language inside the human body can be traced as far back as Ancient Egypt and find their most renowned representatives in Paul Broca and Carl Wernicke in the 19th century (1, 2). What all of them had in common were the lesion-based associations. In other words: the establishment of function was based on brain lesions (observed post-mortem) and the resulting linguistic behavior. An important consequence of this approach was the so-called “localism” or “localizationism,” according to which the brain has centers with specialized functions, and this structure-function organization is rigid and static (1–3). Eventually, other approaches were developed, resulting from both the debate among researchers from different areas and technological advancement. Concerning the neuroscience of language, these new approaches lead, for instance, to new descriptions of language disturbances that were based on linguistic aspects and categories (4–6), instead of physiology alone. Finally, the possibility of taking a look inside the brain allowed researchers to test the relationships they had previously established and to refine their knowledge on brain anatomy and brain function.

The development of neuroimaging techniques not only allowed the observation of living brains, but it also made possible its dynamic recording, to a point where researchers could access both healthy and unhealthy brains, and watch their functioning on-line (1). Due to such technologies, research on neurology and the related fields was expanded beyond clinical application, and turned to the study of the development and the function of the brain (7). However, novel means of experimentation meant novel methodological inquiry. That is the case with the functional magnetic resonance imaging (fMRI). This technique can measure the regional cerebral blood flow (CBF) and changes in the blood oxygenation, the so-called blood-oxygenation-level dependent contrast (BOLD), which provide very accurate spatial information. Because the living brain cannot be shut down for investigation, this real-time access of cerebral activity leads to the observation of several functions simultaneously, and thus to the identification of several different brain areas, regardless of them being crucial or not to the targeted function (3).

When it comes to language, the truth is that the current models are still not complex enough (3, 8). It is unlikely that a single language task is going to encompass every possible language aspect, and it is also unlikely that, when executing a language task, only brain areas that are language-related are going to be activated (7, 9). Analysis of experimental data provides evidence that language processing in the brain is task-dependent, following different paths according to the demand (10, 11), something previously suggested in other fields of cognitive studies (12). Therefore, researchers face a variety of simultaneous processes, not always being capable of distinguishing those that are inseparable from each other from those whose co-occurrence happens by chance.

This matter goes beyond the interpretation of brain images because language is an object of a very particular nature. It has different definitions and approaches for each field or theoretical framework, it has properties that are immeasurable (or unquantifiable), and it is not directly accessible—being only observed through usage. Current methodology for fMRI is careful about the machine setting, about the software for data analysis, and about the proposition of anatomical and physiological correlations; however, it may be less concerned about linguistic context and about the theoretical linguistic references for the phenomena the experiments claim to describe. This tendency for weakly defining some concepts from the Human Sciences is not new and has been frequently debated. They are often taken as self-evident so that their definition may be seen as unnecessary (13).

The aim of this article is then to discuss the current limitations of fMRI experimentation with language, the hardships of working with ill-defined objects, and to which measure linguistic theory can collaborate in both developing better methods for neuroimaging studies, and in refining our treatment of language as a scientific object, in isolation and in combination with fMRI.

## Language as an Object

### Is There an Objective Definition?

What are we talking about when we talk about language? Saussure (14) explains that linguistics is unlike other sciences, with their previously determined objects, because when studying language, the same element may be treated differently depending on how it is considered. The very notion of what is language differs among authors, as does its categorization and the definitions of its units.

Popularly, language may be seen as a communicative system or even a code. Going deeper into linguistic theories, however, different concepts can be found. Saussurian linguistics conceives language as a system of signs, and separates it into langue and parole; the former being the equivalent to competence, or the inner linguistic knowledge of the subject, and the latter being the equivalent to performance, or linguistic behavior (14). Later on, Chomsky approached language as a computational system or an algorithmic grammar. In other words: a limited set of rules from which sentences are created (15, 16). He also mentions a “faculty of language,” considering language “an internal component of the mind/brain” that is universal to the human species (16–18). It is an opposite view to that of Tomasello (19), who, because of his studies about primates, considers that language is not a primary function in itself, being instead a social product of the components of human cognition. It is also possible to use “language” to refer to the “language of thought,” also known as “mentalese.” This makes the term “language” open to interpretation according to the view of the researcher—or the reader.

There are different sorts of experiments with language, which may or may not be dealing directly with one of the different hypotheses about the status of language inside the human brain. However, even those targeting very specific and punctual aspects or units of language must have them clearly defined, because each theoretical framework may encompass a different set of parameters for classifying the linguistic units and their relationships among each other. Terms such as word, speech, and semantics are largely used, even if they are not followed by any sort of explanation. Therefore, a wide range of tasks with the same name exists, even if they are very different among themselves. One may consider, for instance, the notion of “word.” It may be taken as equivalent to the written conventions of each language, or as equivalent to an idea or concept. If the researchers opt for the latter, they must subsequently decide if this concept shall be broken into smaller unities or not. They must also decide if concepts are universal or if they depend on the language. It is then hard to know when designing a lexical experiment, what words they can use, and at what are they exactly looking.

### Context

Everything surrounding human utterances can be classified as “context,” and the importance of each thing is decided according to the researcher's target. Linguistic studies involve fields dedicated to describing and understanding to which measure these extralinguistic information and also the subject's intentions affect meaning (20–22). There are neuroimaging experiments that seemingly reiterate the importance of context, showing how much the activation pattern can vary because of it (23). Some of them will imply that the presence of an extralinguistic context and of an interlocutor may simplify the processing of language (24, 25).

It is hard to objectively access linguistic data, regardless of the definition adopted by the researcher, because the psychological reality of language is not something directly measurable or observable. The sole way of turning it into material with which one can work is transforming it in behavior, which in this case means processing and producing speech (26). But linguistic meaning is not something absolute. So, when this happens, the human brain takes into account multiple types of information, and also put different systems to work (9, 16, 25, 27, 28).

One example of non-linguistic stimuli interfering in the linguistic processing is the McGurk Effect, where visual information and the listener's expectations affect comprehension of an auditory input (28, 29). It has also been noticed that the motor area relative to phonatory articulation is activated when the conditions for speech comprehension are bad (10). Still, other studies have found that semantics and syntax may be mutually dependable for meaning attribution (30–33). Comparing language experiments, it is noticeable that the comprehension of language is not a unique process, that it does not rely on verbal input alone, and that it may occur differently depending on a vast array of variables, such as the task demand, the listener's expectations, and the information available that can be integrated with the speech (10, 27, 34). In other words, it varies depending on the conversational context.

The discussion on the relevance of “context” can still get more complicated. It may be the extralinguistic information, such as the situation in which the utterance occurs, or the background information (23). However, the context may also be purely linguistic, such as a string of sounds, the lexical item preceding/following the targeted unit, or a word's inner composition (11, 31)—and one may refer to those as “linguistic levels” or “levels of computation.” The linguistic information present in a context may augment the brain's response for a certain stimulus because this response can be affected by variables such as morphological structure, lexical category, frequency of the word or the letter combination, or the morphologic or semantic relationships between nearby stimuli (11, 35–40).

### Controlling Language and Its Units

Experiments focused on language often describe part of their methods referencing what they call a “language task,” and then they specify such tasks under different criteria, being mostly:

(a) the target of such a task. For example: fluency, mental imagery, grammar, cloze probability.
(b) the action involved in such a task. For example: word retrieval, lip-reading, grammatical decision/judgment.

Despite describing the task itself and what is expected to be observed through it, those experiments seldom base their definition of linguistic units and aspects in descriptions from linguistic theories, often resulting in vagueness concerning the object. A major downside of this lack of a linguistic theoretical basis is the higher risk of bias. Another possible limitation of such experiments is the subject's very ability to refer to language as language, instead of as its meaning. This ability, referred to in Linguistics as “metalanguage,” is probably the main source of misinterpretation of experimental data. It is mostly dealt with in linguistic studies concerning children and subjects with some type of language impairment, and they are not new. Reports can be found of situations in which the patients failed to produce the required structures during a test, but were able to spontaneously do so once out of it; or situations where the subject was unable to separate linguistic evaluation from contents evaluation (41, 42).

Another relevant obstacle to analyzing linguistic phenomena is the episodic aspect of some data, whose emergence is hardly possible to be caused by the researcher (43, 44). Examples of that are the “tip of the tongue” phenomenon (TOT's) (33), and phonetic or lexical substitutions observed in paraphasias. That means that only case studies can be made of those because the chances of statistical significance would be very low in experiments.

Besides the influence of linguistic levels and other variables in language processing, and the hardships of producing some of the targeted phenomena in experimental conditions, there is still one important discussion to be had, and it is whether or not linguistic properties can be studied in isolation from one another, or if they can be accessed at all. Authors such as Vigotsky would argue that the scientific method is an obstacle to studying higher human psychological activity because even though experiments may shed light on “inferior” human behavior, they cannot contain the “essence” of the more complex ones (45). Chomsky went further, arguing that, despite our ability to theorize about both our linguistic competence and our linguistic performance, it may be impossible to discover how we transform the former into the latter (18).

It is common sense in Linguistics that decisions concerning one level of language do not suffer influence from that level alone. The metalanguage mentioned above, for instance, underlies every action of linguistic judgment. There is still the difference in behavior between content words (such as nouns and adjectives) and functional words (such as articles and prepositions). The choice of the latter depends on meaning, but also grammar and even on phonology. So much so that one of the manifestations of so-called “agrammatic aphasia” (or agrammatism) is the absence or misplacement of such words (37, 46, 47). There is also evidence that the process of syntactic function attribution—parsing (48)—depends equally on semantics and syntax (24, 31). Nevertheless, that does not prevent researchers' efforts to separate the stimuli in different language categories and link those to different brain structures (29). One of the studies trying to do so with semantics and syntax ended up observing an overlap of activation for both (24), something coherent with what many descriptions from Linguistics assert. That presses on the question of whether compartmentalizing language levels is a task in which researchers are going to be successful.

## On fMRI's Application in Language Experimentation

### A Brief Presentation of fMRI's Fundamentals

Functional magnetic resonance is based on evidence that an active area of the body demands more oxygen, leading to an increase in this area's blood flow, which is then detected inside the magnetic field generated by the MR machine (49). This technique provides a series of images where the brain areas are highlighted according to the intensity of its activation, and these images follow the brain changes throughout a time interval. Given the crossing of information of differently oriented magnetic fields, the spatial precision of this method is the highest reached so far, causing it to stand out among the other techniques for brain investigation. Besides, it is a safe method, non-invasive, with no radiation emission, and with few restraints concerning subjects' participation in an experiment.

However, this high precision comes at a cost. The image acquisition takes time, and the subject must stay still throughout the entire process, something which can be uncomfortable. In addition, the limited space and the need for stillness cause a significant restriction in possible types of tasks.

### Compatibility Between Technique and Object

The properties of language as an object are in constant struggle with the need for control intrinsic to the scientific method. An optimal fMRI method would balance precise object definitions, reasonable control of variables, and the complexity of language. However, researchers are not only unable to avoid the interference of other brain functions during the imaging acquisition, but also to find a task that involves all of the linguistic levels at once. Moreover, there are epistemological questions yet to be discussed. For instance, whether every language variation will produce a significant change in the brain activation path; or whether the same utterance will always provoke identical activation. Also, because the fMRI technique presupposes a function to be directly attributed to one or more brain structures, researchers must decide if it is expected that each linguistic unit will trigger a different and specific brain function, and ponder if their units perceived in the brain are equivalent to those pointed out by linguists.

The most basic linguistic structures and concepts are very abstract and very deeply embedded in the human brain to be directly observed. The utterance of a single lexical item is already different from the concept it represents (16). It is also different from the utterance of a whole sentence containing this item, implying that when experimenting with words, one must observe more than only the words. The same goes for sentences, phones, and meaning. As soon as language becomes materialized into speech, it is surrounded by external meanings and intentions.

Moreover, if experiments cannot grasp a “pure” linguistic concept, the same might be asked about its dwelling inside the brain. Previous experiments have already suggested that contextual information may change the activation pattern and/or the intensity of the electrical signals during language processing (10, 23, 25, 50), which is in tune with older linguistic descriptions. Attempting to delineate the exact pathways underlying an utterance is challenging. Firstly, because it requires the sensory-motor system and the conceptual-intentional system in order to be accomplished (17). Secondly, because circumstances that emulate real life scenarios would force our brains to deal with too much information, and that would make it very difficult to control for what is triggering the observed neural behavior.

This is an obstacle to the usual block design employed in fMRI studies, despite its methods of isolating the targeted signals, because they are fit to situations with clearly timed stimuli. Moreover, there is also the physical limitation of the MRI machine's environment that restricts the range of possible activities and the subject's interaction with the external environment. That is why, when it comes to extralinguistic information, techniques such as electroencephalography (EEG) or magnetoencephalography (MEG), that provide a higher degree of freedom for the subject may allow more richness of the environment for the task at hand—one that is closer to real life circumstances (51). Still, those have smaller spatial resolution and are preferred to study how long stimuli take to be processed. So the effect of this sort of information on the processing path remains hard to tell, for the sort of task required to observe it is still hard to be fit inside an MR machine and, even if it could be fit there, there would still exist the issue of the extension of the activation and of how to interpret it.

This leads us to the images themselves and the question of how the correlations we seek should be made. Poeppel and Embick (52, 53) referred to two main issues of the crossing between linguistic theory and neurosciences: the Granularity Mismatch Problem (GMP) and the Ontological Incommensurability Problem (OIP). The former states that the two fields deal with different “elemental components,” one with neurons and brain regions, one with concepts and language unities. The latter states that these elemental components cannot be matched. In other words, these fields develop independently to a point where no solid link can be made between them. They even argue that the linguistic theory allows descriptions of language with a level of detail that neuroscientists might not be able to translate into physiological measurements—which would be equivalent, for instance, to matching single neurons to single words, concepts, or structures.

That is probably the most challenging question left for linguists, if not neuroscientists. The knowledge of the relationship between brain structures and language processing is apparently affected by the descriptions from linguistic theory. Nonetheless, even if an optimal method for language experimentation existed, we would still need to develop a way of translating neurobiological information into linguistic descriptions.

## Final Considerations

Neuroimaging technologies allowed science to break through the localist hypotheses from the previous centuries, but now their limitations are being re-evaluated. The adequacy of the fMRI method for studying the higher functions of the brain may be questioned, because of all of the restrictions it imposes on the subject's activity. However, it must be said that the need to study language processing in spontaneous situations is not a consensus. Some authors argue that, despite the clear difference between the experimental contexts and the spontaneous use contexts, the basic processes underlying the linguistic activity remain the same, and there is no need for revamping the experimental conditions (53). The problem is that there seems to be no methodological unity among the language experiments performed in different research areas. Whereas linguistic theories are mostly based on behavioral data, neuroscientific ones are mostly based on anatomical and physiological data. One does not take the other as a parameter for checking results or for outlining research strategies. Moreover, the studies may or may not employ linguistic theory in describing the language phenomena they are observing, and this lack of precision may be generating vague data. Therefore, even though the current experiments tell us something, it is hard to tell exactly what it is and to what extent their results can be generalized.

The knowledge of the language and the brain has changed a lot through time; it now points to a high integration among all of the brain areas and to an interdependency among processes, much different from the earlier models of brain functioning. So far, there is research investigating the role of Broca's area during non-linguistic tasks (54), or of the cerebellum in language processing (55, 56), or even of the amygdala in prosody perception (57). This may end up leading to descriptions of new properties and new levels of language. It can also lead to changes in the very concept of what language is. However, answering these questions requires a thorough description of the currently existing linguistic data, and also a thorough discussion on how deep the structure-function correlations can get.

All things considered, the crossing of knowledge from different areas is essential for making the theories more robust. If the neuroimaging results and the linguistic descriptions do not match, it means that pieces of this puzzle are still missing. So, physiological data must have no priority over linguistic theory, because the second has descriptions and predictions just as strict and specific as the former. They must be equally taken into consideration (58).

The changes required for making these experiments better are not all worked out, but some slight improvements are already in reach. The investigation of specific linguistics' concepts is not absent from the neuroimaging research (23, 33, 59). Manners of enriching the context and providing the subject with some background information can already be seen with the presentation of small narratives preceding a target sentence so that the subject can establish references for the components of this sentence; or also with tablets that are specially designed for writing tasks inside an fMRI machine (51). Moreover, recent studies in the field work with the so called “naturalistic stimuli,” based on the use of videos and short movies, that allow for the acquisition of visual information on the interlocutor's gestures and scenario, and the observation of the subject through a more prolonged stimulus (23, 34). These are attempts to counterbalance the limitations of the MRI machine, as they offer multimodal input, and they set an environment that is both more complex and more engaging for the subject (34, 60). However, these new developments still need working and validation, because researchers are still finding the patterns and models that fit each situation and each target, and how these different stimuli affect the neural networks. Concerning the risk of bias, even simpler tasks can be improved if the stimuli are properly chosen, based on linguistic levels and categories. This can be accomplished, for example, by controlling the words of a list for the lexical category, morphological and phonetic structure, semantic field, or also frequency.

It is true that researchers do not even know if the brain is going to react to the same language categories established from speech studies. This uncertainty is one of the things making the experimental results in this area of research vague, and it may be going to follow each new development in the field until these details are addressed. Studies are achieving interesting and clarifying results when trying to approach linguistic concepts through neuroimaging technology, some of those corroborating today what linguists have hypothesized a long ago, thus clearing the pathway for new theories and discussions in linguistics. Much can be expected from a balanced association between these two fields, and the main contribution to neuroscience might be exactly the bigger representativity in their studies, and the end of ambiguities or vagueness surrounding most of the current experimental data.

## Author Contributions

MA and FC contributed for the conception and the design of the study. MA organized the database and wrote the first draft of the manuscript and its sections. FC made the manuscript revision, read, and approved the submitted version. All authors contributed to the article and approved the submitted version.

## Conflict of Interest

The authors declare that the research was conducted in the absence of any commercial or financial relationships that could be construed as a potential conflict of interest.
